# Relation of Serum Copper Status to Survival in COVID-19

**DOI:** 10.3390/nu13061898

**Published:** 2021-05-31

**Authors:** Julian Hackler, Raban Arved Heller, Qian Sun, Marco Schwarzer, Joachim Diegmann, Manuel Bachmann, Arash Moghaddam, Lutz Schomburg

**Affiliations:** 1 Institute for Experimental Endocrinology, Charité-Universitätsmedizin Berlin, Corporate Member of Freie Universität Berlin, Humboldt-Universität zu Berlin, and Berlin Institute of Health, D-10115 Berlin, Germany; julian.hackler@charite.de (J.H.); raban.heller@med.uni-heidelberg.de (R.A.H.); qian.sun@charite.de (Q.S.); 2 Bundeswehr Hospital Berlin, Clinic of Traumatology and Orthopaedics, D-10115 Berlin, Germany; 3 Department of General Practice and Health Services Research, Heidelberg University Hospital, D-69120 Heidelberg, Germany; 4 ATORG, Center for Orthopaedics, Aschaffenburg Trauma and Orthopaedic Research Group, Trauma Surgery and Sports Medicine, Hospital Aschaffenburg-Alzenau, D-63739 Aschaffenburg, Germany; schwarzer27@gmail.com (M.S.); Joachim.Diegmann@klinikum-ab-alz.de (J.D.); manuel.bachmann.md@gmail.com (M.B.); 5 Orthopedic and Trauma Surgery, Frohsinnstraße 12, D-63739 Aschaffenburg, Germany; email@arash.de

**Keywords:** trace element, inflammation, ceruloplasmin, micronutrient, COVID-19

## Abstract

The trace element copper (Cu) is part of our nutrition and essentially needed for several cuproenzymes that control redox status and support the immune system. In blood, the ferroxidase ceruloplasmin (CP) accounts for the majority of circulating Cu and serves as transport protein. Both Cu and CP behave as positive, whereas serum selenium (Se) and its transporter selenoprotein P (SELENOP) behave as negative acute phase reactants. In view that coronavirus disease (COVID-19) causes systemic inflammation, we hypothesized that biomarkers of Cu and Se status are regulated inversely, in relation to disease severity and mortality risk. Serum samples from COVID-19 patients were analysed for Cu by total reflection X-ray fluorescence and CP was quantified by a validated sandwich ELISA. The two Cu biomarkers correlated positively in serum from patients with COVID-19 (R = 0.42, *p* < 0.001). Surviving patients showed higher mean serum Cu and CP concentrations in comparison to non-survivors ([mean+/−SEM], Cu; 1475.9+/−22.7 vs. 1317.9+/−43.9 µg/L; *p* < 0.001, CP; 547.2.5+/−19.5 vs. 438.8+/−32.9 mg/L, *p* = 0.086). In contrast to expectations, total serum Cu and Se concentrations displayed a positive linear correlation in the patient samples analysed (R = 0.23, *p* = 0.003). Serum CP and SELENOP levels were not interrelated. Applying receiver operating characteristics (ROC) curve analysis, the combination of Cu and SELENOP with age outperformed other combinations of parameters for predicting risk of death, yielding an AUC of 95.0%. We conclude that the alterations in serum biomarkers of Cu and Se status in COVID-19 are not compatible with a simple acute phase response, and that serum Cu and SELENOP levels contribute to a good prediction of survival. Adjuvant supplementation in patients with diagnostically proven deficits in Cu or Se may positively influence disease course, as both increase in survivors and are of crucial importance for the immune response and antioxidative defence systems.

## 1. Introduction

The immune system relies on a sufficiently high supply of micronutrients in order to fulfil its essential functions in surveillance and defense. Certain vitamins and trace elements are assumed to play a key role in coping with the coronavirus disease (COVID-19) caused by the severe acute respiratory syndrome coronavirus 2 (SARS-CoV-2), including vitamin D and C, along with the essential trace elements copper (Cu), selenium (Se) and zinc (Zn) [1,2]. Despite a number of insightful and thought-provoking recent reviews and hypotheses on the potentially underlying mechanisms of interrelation, laboratory analyses and clinical studies are few and the respective database is limited.

The inconclusive knowledge is probably reflected best in the discussions on a potential role of vitamin D in COVID-19 [3,4,5]. While there is some consensus that vitamin D concentrations are decreased, it is unclear whether supplementation would be beneficial [6,7,8]. The observed deficiency in COVID-19 may result from its downregulation in response to systemic inflammation [9].

The same applies to the essential trace elements Se and Zn, where supplementation studies are suggested in order to correcting the observed deficiencies in COVID-19 [10,11,12]. The rationale for this hypothesis is mainly based on two fundamental findings, i.e., the general notion of a dysfunctional immune system in Se or Zn deficiency [13,14], and a depressed Se or Zn concentration observed in inflammatory or severe diseases [15,16,17]. The detected decline in the circulating concentration of Se and Zn may reflect a meaningful adaptation of the organism to the infection as part of the anti-viral response, but it is also of relevance to target tissues relying on a sufficiently high supply. Consequently, both circulating and target tissue deficits may result, collectively causing a depressed trace element status, whereby “status” denotes a concept encompassing intake, metabolism and reserves of an essential micronutrient [18,19]. As intracellular and circulating concentrations are interrelated and the latter only are accessible for analytical monitoring, circulating parameters are established as biomarkers of trace element status [20,21,22,23]. The Se transporter selenoprotein P (SELENOP) along with glutathione peroxidase-3 and total serum Se concentrations are established biomarkers of Se status [22,23,24], whereas serum Cu along with ceruloplasmin (CP) concentrations serve as readily available biomarkers of Cu status [25,26,27].

In the case of Se, COVID-19 cure rates were associated with Se status in different populations of China with varying habitual intake levels [28], and disease severity along with mortality risk were directly correlated to Se deficiency in laboratory analyses of individual patients [29,30,31]. Together with the knowledge on an increased mutation rate of virus species in a Se-deprived host organism [32], and the generally increased risk of disease-related death in Se deficiency [20], it appears prudent to avoid Se deficiency as a meaningful preventive measure. Whether supplemental Se will yield positive health effects in COVID-19 remains to be studied. The data base on Zn in COVID-19 is similarly limited, and low Zn concentrations are observed in severely diseased patients [33,34]. In contrast to the declining Se status in non-surviving patients, serum Zn levels seem to recover in COVID-19 during the hospital stay, indicative of a redistribution between circulation and immune cells [29].

Similar to Se and Zn, the essential trace element Cu is also needed for a regular immune response. A number of catalytically active cuproenzymes affect general developmental as well as metabolic and adaptive pathways [35,36]. Accordingly, a systemic deficiency in Cu is associated with a number of diverse symptoms that are related to Cu-containing enzymes, e.g., incomplete collagen formation, pigmentation defects, catecholamine misbalance and impaired neuronal signalling as well as muscle weakness and cardiomyopathy, partly due to insufficient biosynthesis of lysyl-oxidase, dopamine beta-hydrolase and cytochrome c oxidase, respectively [37,38,39]. In addition, severe hematologic and neurologic symptoms develop under conditions of chronic Cu deficiency [40,41,42]. Inherited defects in key genes of Cu transport cause childhood-onset and potentially fatal Cu deficiency with severe and life-threatening neurological defects, as observed in Menkes disease with mutations in the intracellular Cu-transporter *ATP7A* [43]. Under regular conditions, severe changes in Cu metabolism are observed in response to inflammation, where serum Cu and CP behave as positive acute phase reactants, i.e., in opposite direction to serum Se and SELENOP [44,45,46]. For these reasons, we decided to determine Cu and CP in relation to biomarkers of Se status in serum of patients with COVID-19, and to test their value for predicting survival odds.

## 2. Materials and Methods

### 2.1. Study Design

Longitudinal serum samples were available from a cross-sectional study of surviving and non-surviving patients with COVID-19, who were hospitalized at Klinikum Aschaffenburg-Alzenau in Germany, as described earlier [30]. The study was conducted in accordance with the Declaration of Helsinki. Ethical counselling had been obtained from the authorities in Bavaria, Germany (Ethik-Kommission der Bayerischen Landesärztekammer, EA No. #20033), and the study was registered at the German Clinical Trial Register (Deutsches Register Klinischer Studien, ID: DRKS00022294). All patients enrolled or next of kin provided written informed consent. On average, five consecutive blood samples were collected per patient and were available for analysis. Serum was prepared, stored at −80 °C, and sent on dry ice to a remote lab from the clinics for Cu status analysis. The analytical measurements were done by scientists blinded to disease information. Reference values were derived from adult subjects enrolled in the European Prospective Investigation into Cancer and Nutrition (EPIC) study, who had been analysed by the same technology as published earlier [47,48].

### 2.2. Serum Copper Analysis

Serum Cu concentrations were extracted from the trace element spectra obtained during total reflection X-ray fluorescence (TXRF) analysis of the patients’ serum samples using a benchtop TXRF spectrometer (S4 T-STAR, Bruker Nano GmbH, Berlin, Germany), as described [30]. To this end, the samples had been diluted with a gallium standard, applied to polished quartz glass slides and analysed after drying along with seronorm serum standards (Sero AS, Billingstad, Norway). The Cu concentrations measured were within the specified range of the standard, and the inter-assay coefficient of variation (CV) was below 10% at a Cu concentration of 1691 µg/L serum.

### 2.3. CP Quantification by ELISA

Circulating CP concentrations were determined directly from serum by a recently developed sandwich method with monoclonal antibodies (mAb) to human CP as described [27]. Briefly, serum of COVID-19 patients was pre-diluted 1:300, and aliquots of 50 µL were incubated for 30 min at room temperature on sandwich ELISA plates pre-coated with a CP-specific mAb (mAb1). Quality of measurements was verified by using a commercially available human CP standard preparation (Ceruloplasmin 187-51-10, Lee Biosolutions, Maryland Heights, MO, USA). A three-times automatic wash step was performed to rinse the ELISA plates using a HydroFlex^TM^ microplate washer (Tecan Group AG, Maennedorf, Switzerland). For sandwich detection, 50 µL of a CP-specific mAb-HRP conjugate (mAb2) was incubated for 30 min. Unbound mAb2 was rinsed and the enzymatic detection was started by adding 100 µL of 3,3′,5,5′-tetramethylbenzidine (TMB). The reaction was terminated by sulphuric acid (0.25 M, 100 µL per well). Spectrophotometric read out was recorded within 10 min at 450 nm using a NanoQuant Infinite 200 Pro microplate reader (Tecan Group AG).

### 2.4. Statistical Analysis

Statistical analyses were conducted by using the language and environment for statistical computing R, version 4.0.3. The analytical packages *tidyr*, *dplyr*, and *pROC* [49] were used along with the graphic package *ggplot2* [50]. The Shapiro–Wilk test was used for assessing the normal distribution of values. Correlations were tested by Spearman correlation analysis. Comparisons of the characteristics between two groups were conducted by Mann-Whitney-U test, more than two groups were compared with Kruskal-Wallis test.

As this is an exploratory post-hoc analysis, all p-values are to be interpreted descriptively, and no adjustment for multiple testing was adopted. Variable selection was performed via stepwise AIC selection [51,52]. Differences between ROC curves were assessed by the DeLong’s test [53]. All statistical tests were two-sided, and *p*-values < 0.05 were considered significant; * *p* < 0.05, ** *p* < 0.01, *** *p* < 0.001, and **** *p* < 0.0001.

## 3. Results

### 3.1. Characteristics of Patients

A total set of n = 173 consecutive serum samples from n = 35 hospitalized patients with PCR-proven SARS-CoV-2 infection and health symptoms of COVID-19 were available for analysis. Basic anthropometric information is provided below. The majority of the subjects and samples analysed overlaps to the study published on changes in Se status during COVID-19 [30]. Notably, the group of non-survivors was significantly older than the patients who could be successfully discharged from the hospital after an average time span of 16 days (Table 1).

### 3.2. Copper (Cu) Status Analysis and Comparison to Serum Se Status

**Figure 1:**
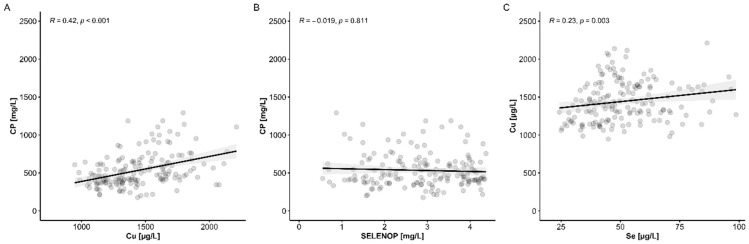
Analysis of serum Cu status in COVID-19 in relation to serum Se status. (**A**) The two biomarkers of Cu status, i.e., total Cu and CP concentrations showed a significant positive correlation in the full collection of serum samples. (**B**) In comparison, there was no interrelation of serum CP with SELENOP, whereas (**C**) serum Cu and Se showed a significant positive correlation with a marginal slope. *R*: Spearman correlation coefficient (2-sided, 2-tailed), *p*-values are indicated.

Cu status was evaluated from all serum samples available by two complementary biomarkers, i.e., total serum Cu and CP concentrations. The two biomarkers showed a significant positive and linear correlation over the full concentration range, supporting the assumption of a high quality of the available clinical samples and the analytical test systems used, and of the suitability of both parameters as complementary biomarkers of the endogenous Cu status. (Figure 1A). The correlation of serum Cu and CP was of medium strength (*R* = 0.42) and displayed a considerable slope. Total serum CP and SELENOP showed no significant interrelation (Figure 1B), whereas serum Cu and Se concentrations correlated positively, albeit with a marginal slope only (Figure 1C).

### 3.3. Cu Status of COVID-19 Patients in Relation to Survival

**Figure 2:**
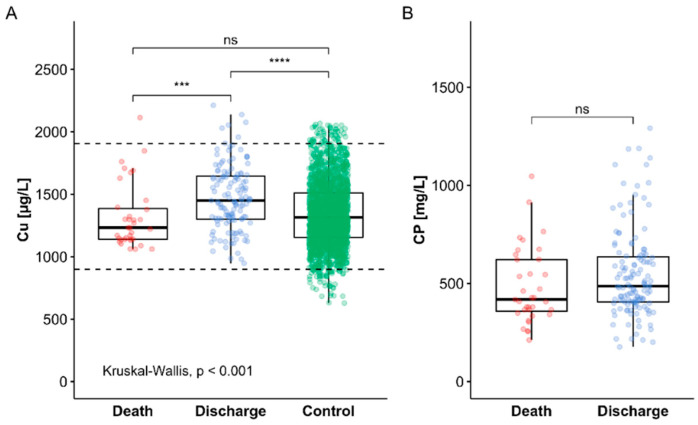
Elevated serum Cu status in patients surviving COVID-19. Serum samples from surviving and non-surviving COVID-19 patients were analysed for two biomarkers of Cu status, i.e., total serum Cu and CP concentrations. (**A**) Samples from COVID-19 survivors displayed significantly elevated serum Cu in comparison to non-survivors and to a reference cohort of healthy adult subjects. (**B**) Circulating levels of the Cu transport protein CP were slightly but not significant elevated in surviving as compared to non-surviving patients. Comparisons between two groups by Mann-Whitney U test, and among the groups by Kruskal–Wallis test; *** *p* < 0.001 and **** *p* < 0.0001.

An average population-wide reference range for serum Cu concentrations in healthy subjects was deduced from the data obtained earlier from the cross-sectional EPIC study [47,48]. According to the 2.5th–97.5th percentile of the data, the reference range for total serum Cu concentration was 897.8–1906.0 µg/L. None of the serum samples analysed was below, and only few patient samples showed serum Cu slightly above the reference range of Cu concentrations (Figure 2A). Notably, almost all of the elevated Cu levels were detected in samples from patients surviving COVID-19. The direct comparison reveals that the samples from the group of non-survivors was not different from the reference cohort, whereas the surviving patients showed on average elevated serum Cu levels in comparison to non-survivors and in comparison to the reference range (Figure 2A).

Our prior analyses of subjects enrolled into the large EPIC cohort was unfortunately not including measurements of human CP, as the analytical assay for this important parameter of Cu status was not yet established in our lab at the time of study. For this reason, an assessment of the CP levels was restricted to the direct comparison of CP concentrations in serum samples from surviving versus non-surviving patients. On average, a tendency but no significant difference in serum CP status was observed when comparing the groups of patients in relation to survival (Figure 2B).

### 3.4. Dynamics of Serum Cu Status in COVID-19 in Comparison to Se Status and in Relation to Survival

**Figure 3:**
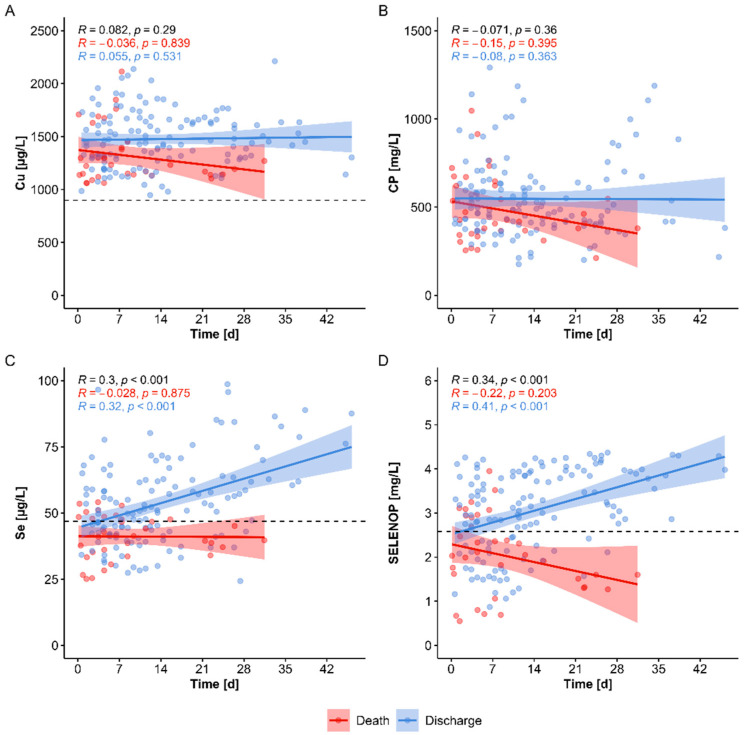
Comparison of dynamic changes in biomarkers of Cu and Se status in relation to survival. Serum samples from different time points after hospital admittance were available form surviving (blue) and non-surviving (red) COVID-19 patients. (**A**) Total serum Cu concentrations showed no obvious alterations over time during the hospital stay, and were only slightly different between COVID-19 survivors and non-survivors. (**B**) Significant alterations over time were not observed in serum CP concentrations of patients with COVID-19. The stability in Cu status is different to the dynamic changes observed before in the biomarkers of Se status, where (**C**) total serum Se and (**D**) SELENOP concentrations recovered during hospital stay in surviving patients only. Thresholds for deficiencies (broken lines), Spearman correlation coefficients (*R*) and *p*-values are indicated.

Serum samples from COVID-19 patients were taken and collected from time of admittance to the hospital until time of discharge or death. Separating the samples according to survival, differences in serum trace element status can be determined with time in the patients who were dismissed from hospital versus those who died. The direct comparison reveals no significant differences over time in survivors versus non-survivors, neither with respect to total serum Cu concentrations (Figure 3A), nor in relation to circulating CP levels (Figure 3B). These findings are in contrast to biomarkers of Se status. The alterations in serum Se and SELENOP are presented here for direct comparison, i.e., increasing concentrations of total serum Se along with increasing SELENOP concentrations with time are observed in survivors only (Figure 3C,D), as reported before [30]. The overall picture indicates a particular dynamic up-regulation of biomarkers of Se status in surviving patients, whereas biomarkers of Cu status remained relatively constant with time in COVID-19 (Figure 3A–D).

### 3.5. Predictive Value of Compound Biomarkers inclucing the Cu Status for Surviving COVID-19

**Figure 4:**
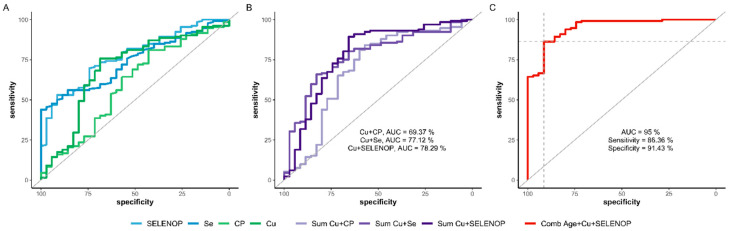
Receiver operating characteristics (ROC) analyses of Cu and Se status biomarkers in relation to survival or death from COVID-19. (**A**) Overview on ROC analyses as univariate prediction models for risk of death based on the serum concentrations of the biomarkers of Cu and Se status, i.e., based on serum Cu (green), CP (light green), Se (blue) and SELENOP (light blue) in isolation. (**B**) Overview on the predictive value of combined markers consisting of both Cu status biomarkers (Cu and CP, light violet) in comparison to mixed markers of Cu and Se status, i.e., Cu and Se (pale violet) and Cu and SELENOP (dark violet), respectively. (**C**) The final biomarker of serum Cu and SELENOP along with age outperformed the other combinations and correctly predicted non-survival with an area under the curve (AUC) of 95.0%. The relative performance is indicated in relation to a non-informative biomarker at the diagonal line at an AUC of 50%.

Finally, receiver operating characteristic (ROC) curve analyses were conducted to assess the potential value of Cu and Se status biomarkers for improving prediction of survival in COVID-19 (Figure 4A–C). To this end, serum Cu as well as CP concentrations were tested alone and in combination with biomarkers of Se status in combination with age of the patients by a stepwise Akaike information criteria (AIC) selection process. The results indicate that a compound biomarker of serum Cu and SELENOP concentrations along with age provides some reliable information on COVID-19 course and survival odds, and outperformed other variables as well as combinations thereof, yielding an area under the curve (AUC) of 95.0. The cutpoint according to the Youden’s J statistics is characterized by a sensitivity of 86.4% and a specificity of 91.4%, indicating a useful biomarker that may contribute to a better assessment of survival chances in COVID-19 (Figure 4C).

This notion is further underlined by the specific characteristics of the models used (Table 2).

## 4. Discussion

This study characterises changes in total serum Cu and CP concentrations, as biomarkers of Cu status, of patients with COVID-19 in relation to survival and disease progression during hospitalisation. As expected, both biomarkers showed a linear and positive correlation in the full group of samples analysed, in line with the notion that CP constitutes the major circulating Cu-binding protein in humans [54,55]. However, neither a strong elevation nor any characteristic kinetics with disease course were observed, and the hypothesized inverse regulation of Cu to the decreasing Se status during hospital stay was not detected. This finding was unexpected, as both serum Cu and CP are known as acute phase reactants, positively correlating to inflammation [56,57], whereas serum Se and SELENOP are established negative acute phase reactants [58,59]. A moderately elevated mean Cu level was present specifically in the group of survivors, potentially indicating a meaningful, health supporting and regular response to the infection, which was not observed in non-survivors. The elevated Cu status concurs with a recovering Se status in survivors [30], and seems to indicate high chances for successful convalescence.

Clinical data on serum Cu status in patients with COVID-19 are few. Pregnant women with COVID-19 have been reported to display a trimester-dependent increase in serum Cu concentrations, with small deviations only in comparison to healthy control pregnancies [34]. Interestingly, serum Cu was elevated in the pregnant women with COVID-19 specifically in the first and third trimester, but not in the middle of pregnancy [34]. Whether these dynamics constitute a positive and pregnancy-supporting response is unknown at present. A very recent analysis of full blood trace elements in COVID-19 from Wuhan, China, indicated a generally increased Cu status in the more severely diseased patients, without observing a difference in full blood Cu when comparing survivors and non-survivors [60]. However, the difference in blood Cu concentration in relation to severity of COVID-19 was small, i.e., in the range of 10% only, and again not compatible with a vivid and strong positive acute phase response to the infection. This notion is supported by a relatively unchanged Cu status in COVID-19 during the hospital stay, as observed both in the analysis of full blood in the study from Wuhan [60], and in our present analysis of serum Cu and CP in German patients.

Despite the relatively stable levels of the Cu status biomarkers over time in hospital, our analysis indicates a higher Cu level in the group of surviving patients with particular relevance for outcome prediction, when combined with the patient’s age and SELENOP status. This finding points to a complex disease-dependent regulation of Cu and Se metabolism, different from what would be expected from severe inflammation alone. The positive linear correlation of serum Cu and Se observed is peculiar, as Se and SELENOP decline in infection [45], whereas Cu and CP increase [46].

The hypothesis of a strong systemic acute phase response of serum biomarkers may not always apply to the patients, as COVID-19 constitutes a slowly developing disease proceeding in several consecutive disease steps, with local inflammation first and eventually worsening respiratory and systemic symptoms later [61]. Strongly elevated inflammation, also called hyperinflammatory syndrome or cytokine storm, has been shown to characterize those patients with highest mortality risk in COVID-19 [62]. Accordingly, the pro-inflammatory cytokine IL-6 as well as hypoxia were expected to interact by synergistically inducing hepatic CP biosynthesis [63,64] and in parallel by suppressing hepatic SELENOP secretion [65,66]. A combined biomarker as ratio of SELENOP over CP, as suggested for assessing thyroid hormone activity in hepatocytes [67], would then provide a most sensitive estimate for survival odds. Yet, our results indicate that at least for the hepatic acute phase reactants CP and SELENOP, the regulation is more complex than expected, as no consistent inverse regulation over time is observed. Either there are more important confounders affecting both transport proteins, or the postulated hypoxia and hyperinflammatory syndrome was not present in the non-survivors analysed, or the missing acute phase response detected via SELENOP and CP is indicative of a failing liver. In view that the hepatic biosynthesis of both CP and SELENOP is also stringently regulated by thyroid hormone [67,68], it may be speculated that the unexpected lack of inverse regulation may be related to critical illness and the euthyroid sick or low-T3 syndrome that may develop in severely diseased COVID-19 patients [69].

This would also be compatible with an elevated oxidative stress and lipid peroxide status, as recently reported from critically ill COVID-19 patients with severe pneumonia who presented with a particularly increased Cu to Zn ratio in face of decreased levels of circulating antioxidants, e.g., vitamin C, Se, glutathione and thiol proteins [70]. In this analysis, the strongest and most significant correlation among all the different biomarkers of oxidative stress was observed for total Cu and lipid peroxides, highlighting a profound impairment of the protective antioxidant defense system in the severely diseased COVID-19 patients, and suggesting the high relevance of a balanced trace element status for coping with the adverse infection sequelae [70]. Like in our study, however, the results are from an observational analysis, not permitting to tell cause from consequence. Yet, the biochemical interrelation of oxidative damage with the trace elements implies that the differences observed are likely intertwined, with a deficiency impairing the immune response and antioxidative defense, which in turn causes further trace element dysbalance and tissue damage. Accordingly, a recent meta-analysis of more than 3400 COVID-19 patients indicated a prime importance of the liver for survival, and highlighted increased mortality risk with elevated circulating liver injury markers (alanine aminotransferase, aspartate aminotransferase, albumin and bilirubin) [71]. These findings are in line with the trace element alterations observed in our study, and more comprehensive analyses are needed next to better decide on the most informative biomarkers or biomarker combinations for improving patient care and early identification of patients with critical disease course.

Among the strengths of the current study is the longitudinal collection and analysis of samples covering the time span from hospital admission to discharge or death, in combination with the assessment of two complementary biomarkers of Cu status, i.e., total serum Cu and CP. However, CP was assessed as total protein concentration only, without quantifying its enzymatic activity. The direct comparison to our prior study on the interrelation of COVID-19 with biomarkers of Se status allows a direct comparison and time-resolved view on these liver-derived acute phase reactants in survivors and non-survivors. Unfortunately, additional parameters of the thyroid hormone axis, oxygen status or inflammation have not been systematically recorded in the patients analysed, and the group of patients was relatively small. Moreover, the nature of our study is observational and as such not suited for deducing causal effects. Nevertheless, the data obtained provide a congruent picture on the Cu status in COVID-19 and its relation to disease course, and suggests that the combined analysis of serum Cu and Se status provides prognostic information on survival odds.

## Figures and Tables

**Table 1:** Characteristics of the COVID-19 patients and serum samples contributing to this study.

	Death	Discharge	Total	Samples
Sex				
Female	5 (71.4%)	15 (53.6%)	20 (57.1%)	116 (67.1%)
Male	2 (28.6%)	13 (46.4%)	15 (42.9%)	57 (32.9%)
Age				
Median (IQR)	89 (81, 94)	69 (38, 91)	77 (38, 94)	
Time to discharge or death [d]				
Median (IQR)	8 (3, 33)	20 (4, 47)	16 (3, 47)	

**Table 2:** Specific characteristics of the predictive models used. For each model, the variable estimates included in the calculations are provided with their corresponding confidence interval (CI).

	Age	Cu	SELENOP	Age + Cu + SELENOP
Age	−3.59 *			−6.79 ***
	[−6.44, −0.74]			[−9.49, −4.09]
Cu		0.70 **		0.97 *
		[0.24, 1.15]		[0.27, 1.66]
SELENOP			1.09 ***	1.61 ***
			[0.64, 1.54]	[0.75, 2.46]
N	35	167	167	167
AIC	23.3	164.9	147.5	82.8
BIC	26.4	171.1	153.8	98.4
Pseudo R^2^	0.57	0.10	0.24	0.70

All continuous predictors are mean-centered, scaled by 1 SD. *** *p* < 0.001; ** *p* < 0.01; * *p* < 0.05.

## Data Availability

The data presented in this study are available on request from the corresponding author. The data are not publicly available due to data safety reasons.
